# Baicalein ameliorates pristane-induced lupus nephritis via activating Nrf2/HO-1 in myeloid-derived suppressor cells

**DOI:** 10.1186/s13075-019-1876-0

**Published:** 2019-04-25

**Authors:** Dan Li, Guoping Shi, Jiali Wang, Dongya Zhang, Yuchen Pan, Huan Dou, Yayi Hou

**Affiliations:** 1 The State Key Laboratory of Pharmaceutical Biotechnology, Division of Immunology, Medical School, Nanjing University, No.22 Hankou Rd., Gulou District, Nanjing, 210093 Jiangsu People’s Republic of China; 2 Department of Rheumatology and Immunology, Nanjing Drum Tower Hospital, The Affiliated Hospital of Nanjing University Medical School, Nanjing, 210008 People’s Republic of China; 3 Jiangsu Key Laboratory of Molecular Medicine, Nanjing, 210093 People’s Republic of China

**Keywords:** Baicalein, Lupus nephritis, MDSCs, Nrf2/HO-1 signal, NLRP3 inflammasome

## Abstract

**Introduction:**

Lupus nephritis (LN) is a representative manifestation in systemic lupus erythematosus (SLE). Some studies have shown that myeloid-derived suppressor cells (MDSCs) play a vital role in the regulation of the SLE process. MDSC infiltration in the kidney as well as inflammation and oxidative stress provokes the acceleration and deterioration of LN. Nuclear factor E2-related factor 2 (Nrf2) is thought to be a major regulator of the antioxidant response. Baicalein is a flavonoid with known anti-inflammatory effects and antioxidant response. However, the effects of baicalein on MDSCs, inflammation, and oxidative stress are not evaluated in the development of pristane-induced LN in mice.

**Methods:**

The renoprotective effect of baicalein was detected in a pristane-induced lupus mice model. NLRP3 inflammasome activation and NF-κB phosphorylation as well as reactive oxygen species (ROS) production and Nrf2 activation were examined. The percentages and function changes of MDSCs were measured. The possible mechanisms of the underlying effects of baicalein on ROS production and signaling pathways of Nrf2/heme-oxygenase (HO)-1, NLRP3 inflammasome, and NF-κB phosphorylation in lipopolysaccharide (LPS)-primed MDSCs were analyzed.

**Results:**

Baicalein reduced proteinuria and attenuated renal function impairment and renal histopathology including intrinsic cell proliferation, cellular crescents, and podocyte injury as well as glomerulonephritis activity in lupus mice. Moreover, baicalein downregulated the activation of NLRP3 inflammasome and levels of ROS or NF-κB phosphorylation, and it enhanced Nrf2 activation. Of note, baicalein inhibited the expansion of MDSCs and improved the function of MDSCs in lupus mice. Through analyzing LPS-primed MDSCs in vitro, baicalein was found to exhibit cytoprotective effects coincident with the induction of Nrf2/HO-1 signaling and the suppression of the NLRP3 inflammasome.

**Conclusion:**

The data show that baicalein alleviates the symptoms of pristane-induced LN and suggest that the alleviation may be attributed to inhibition of MDSC expansion and regulation of the balance of the Nrf2/HO-1 signal and NLRP3 expression in MDSCs.

**Electronic supplementary material:**

The online version of this article (10.1186/s13075-019-1876-0) contains supplementary material, which is available to authorized users.

## Introduction

Systemic lupus erythematosus (SLE) is a typical systemic autoimmune disease, characterized by chronic inflammation and immunological abnormalities. Lupus nephritis (LN) is a representative manifestation in SLE [1]. Approximately 25–50% of SLE patients are suffering from LN, which displays the high expression of inflammatory cytokines, glomerulonephritis, and impaired renal function [2]. Podocytes play a key role in glomerular filtration and the preservation of renal function. Proteinuria is one of the major characteristics of LN, and many LN patients possess the symptoms of podocyte injuries [3]. SLE-like mouse models may develop spontaneously or be induced. Some research have shown that pristane-induced lupus mice are a valuable tool for exploring the multiple mechanisms involved in systemic autoimmunity. Unlike other autoimmune and inflammatory experimental models, the pristane-induced lupus mice mostly are similar to human SLE [4, 5]. Therefore, the pristane-induced lupus mice were selected to explore the pathogenesis of LN.

Myeloid-derived suppressor cells (MDSCs) are defined as a heterogeneous population of immature cells derived from myeloid progenitors, which confer immune-suppressive functions [6]. Murine MDSCs are characterized by the expression of cell surface makers CD11b and Gr-1, which can be sorted into CD11b^+^Ly6C^low^Ly6G^+^ granulocytic MDSCs (G-MDSCs) and CD11b^+^Ly6C^+^Ly6G^−^ monocytic MDSCs (M-MDSCs) [6, 7]. Some studies showed that MDSCs were involved in the disease course of SLE. The treatment of MDSCs derived from C57BL/6 mice reduced the levels of anti-dsDNA antibody in serum and proteinuria in the kidney in the lupus mice [roquin (san/san) mice] [8]. MDSCs inhibited the cytokine-mediated differentiation of naïve B cells into plasma cells and relieved the injuries of the kidney [9]. While other studies suggested that MDSCs were pro-inflammatory and could promote the disease progress in chronic inflammation conditions, Wu H et al. reported that the increased MDSCs in peripheral blood of patients are positively correlated with SLE and MDSCs promoted Th17 polarization by secreting Arg-1 in vitro [10]. In our previous research, we have found that MDSCs promoted Th17 polarization and inhibited Treg differentiation by IL-1β secretion to promote MRL/lpr lupus mice progress [11]. Meanwhile, we also found that MDSCs induced podocyte injury through increasing reactive oxygen species in lupus nephritis [12] and INK128 downregulated the expansion of MDSCs by inhibiting the mTOR pathway to relieve pristane-induced lupus development [13]. As is well known, MDSCs worked as regulators to monitor immune balance partly by the upregulation of ROS, Arg-1, and NO [14], but ROS also damaged proteins and nucleic acids and enhanced inflammation [15]. However, the involvement of MDSCs in LN progression is unclear.

Mounting evidence indicates that oxidative stress is involved in the pathogenesis of SLE [16]. The superfluous production of ROS may impair the antioxidant capacity on exposure to harmful stimuli. Of note, the activated transcription factor Nrf2 possesses an antioxidant role with cellular protection and avoidance of tissue injury [17–19]. Studies also revealed that the expressions of Nrf2 and its downstream molecules, heme-oxygenase-1 (HO-1) and glutathione peroxidase (GPx), were declined in mouse models of diabetic nephropathy and IgA nephropathy [20–22]. Moreover, some studies supported the relationship between oxidative stress and inflammation [23, 24]. NLRP3 inflammasome is an important member of NOD-like receptor (NLR) family. Lipopolysaccharide (LPS) and ROS can induce NLRP3 inflammasome activation and assembly [25]. After activation, NLRP3 can promote the activation of caspase-1, which cleaves inactive pro-IL-1b and pro-IL-18 into their mature forms (mIL-1β and mIL-18) and initiates inflammation. Meanwhile, NF-κB inhibitors protect MRL/lpr mice from nephritis by inhibiting NLRP3 and IL-1β expression [26]. Therefore, a hypothesis is proposed that regulating inflammation and oxidative damage of MDSCs in LN may be a new therapeutic strategy.

Baicalein is a member of the flavonoid family and derived from the roots of *Scutellaria baicalensis Georgi*, which is a traditional Chinese herbal medicine. The antioxidant and anti-inflammatory effects of flavonoids [27] were reported, and baicalein owned numerous bioactivities and pharmacological properties [9, 28–31]. Baicalein reduced oxidative stress in CHO cell cultures [32] and protected human vitiligo melanocytes from oxidative stress through the activation of the Nrf2 signaling pathway [33], suggesting that baicalein might inhibit the development of pristane-induced LN in mice. On the basis of these findings, we hypothesize that baicalein may activate Nrf2 anti-oxidative signaling and suppress inflammation of MDSCs to relieve the development of pristane-induced LN in mice.

In the present study, we found that baicalein reduced proteinuria and attenuated renal function impairment and renal histopathology in lupus mice. Baicalein decreased activation of NLRP3 inflammasome and levels of ROS or NF-kB phosphorylation, but it enhanced Nrf2 activation. Baicalein inhibited the expansion of MDSCs and improved the function of MDSCs in lupus mice. Baicalein exhibited cytoprotective effects coinciding with the induction of Nrf2/HO-1 signaling and the suppression of the NLRP3 inflammasome. These data suggested that the alleviation of the development of pristane-induced LN in mice might be attributed to the inhibition of MDSC expansion and regulation of the balance of Nrf2/HO-1 signal and NLRP3 expression in MDSCs.

## Material and methods

### Mice

Female BALB/c mice (6–8 weeks old, *n* = 7/group) were brought from the Model Animal Research Center of Nanjing University (Nanjing, China) and were housed in a pathogen-free condition under a 12-h light and dark cycle. All procedures involving in mice were approved by the institutional guidelines for animal care and used according to the Animal Care Committee at Nanjing University. All mice were acclimatized for 2 weeks before experiments, then received a single intraperitoneal (i.p.) injection of 0.5 ml pristane or phosphate-buffered saline (PBS), and monitored for the following 7 months. Mice were treated with pristane for 5 months, followed by daily i.p. treatment with or without baicalein (25 mg/kg or 100 mg/kg prepared in PBS) for another 2 months. In the following experiments, the mice were humanely sacrificed after 7 months. The spleen, bone marrow (BM), kidney and blood were harvested.

### Reagents and antibodies

Fluorescein isothiocyanate (FITC)-conjugated anti-mouse CD11b mAb, Allophycocyanin (APC)-conjugated anti-mouse Gr-1 mAb, APC-conjugated anti-mouse F4/80 mAb, FITC-conjugated anti-mouse CD4 mAb, APC-conjugated anti-mouse CD3 mAb, APC-conjugated anti-mouse CD11C mAb, APC-conjugated anti-mouse CD69 mAb, FITC-conjugated anti-mouse MHCII mAb, and FITC-conjugated anti-mouse B220 mAb were obtained from Biolegend (San Diego, CA, USA). Baicalein was procured from Sigma-Aldrich (MO, USA). It was dissolved in 100 mM dimethyl sulfoxide (DMSO) and stored at − 80 °C. Recombinant mouse IL-6, GM-CSF, and MDSC Isolation Kit were purchased from Miltenyi Biotec (Bergisch Gladbaicaleinch, Germany). Tween-20, the oxidation-sensitive dye DCFDA detection Kit, cell lysis buffer for western blot analysis, and phenylmethanesulfonyl fluoride (PMSF) were purchased from Beyotime (Shanghai, China). An Annexin V-FITC Apoptosis Detection Kit was obtained from BD Pharmingen (New Jersey, USA). LPS and ATP were obtained from Enzo Life Science (Farmingdale, NY, USA). Trizol Reagent and SYBR green dye were obtained from Invitrogen. Dimethyl sulfoxide (DMSO), streptomycin, penicillin, RPMI-1640 medium, and fetal bovine serum (FBS) were obtained from Gibco (Grand Island, NY, USA). Antibodies for NLRP3, IL-1β, caspase-1 p20, Histone 3, NF-κB P65, β-actin, Nrf2, and HO-1 were procured from Cell Signal Technology Inc. Antibodies for caspase-1 p45 were obtained from Abcam. Horse-radish peroxidase (HRP)-linked horse anti-mouse IgG and HRP-linked goat anti-rabbit IgG were obtained from Thermo Fisher (MA, USA). A Mouse Albumin ELISA Quantitation Set was purchased from Bethyl Laboratories (NY, USA). Mouse IL-1β, IL-18, IL-6, IL-17, interferon (IFN)-α, and IFN-γ were procured from R&D Systems (Minneapolis, MN, USA). An Anti-Wilms’ Tumor Antibody (WT-1) was obtained from Merck Millipore.

### Generation and isolation of MDSCs

Bone marrow (BM) cells were isolated as described previously [26, 34]. In brief, tibiae and femurs were removed from BALB/c mice and BM cells were flushed. Then BM cells were cultured in the medium supplemented with 40 ng/ml murine IL-6 and 40 ng/ml GM-CSF for 4 days. Spleen-derived MDSCs were purified from pristane-induced lupus mice using an MDSC Isolation Kit.

### ROS detection in MDSCs

ROS production was measured by the oxidation-sensitive dye DCFDA. MDSCs were incubated at 37 °C in an RPMI 1640 medium in the presence of 2.5 μM DCFDA and simultaneously cultured with 1 μg/ml LPS for 30 min. Then cells were washed with 0.5 ml PBS twice and measured by flow cytometry (Becton Dickinson, San Diego, CA, USA).

### Measurement of mitochondrial superoxide generation

Mitochondrial superoxide generation in MDSCs was assessed with MitoSOX Red (Molecular Probes, Eugene, OR, USA). MDSCs were incubated with 5 μM MitoSOX Red at 37 °C for 10 min and then examined using a flow cytometer.

### Pathological evaluation of LN

The kidneys were fixed with formaldehyde, embedded in paraffin, and stained with hematoxylin and eosin (H&E). The slides were read and interpreted in a blinded fashion, grading the kidneys for glomerular inflammation, proliferation, crescent formation, and necrosis. Interstitial changes and vasculitis were also noted. Scores from 0 to 3 were assigned for each of these features and then added together to yield a final renal scores. For example, glomerular inflammation was graded as follows: 0, normal; 1, few inflammatory cells; 2, moderate inflammation; and 3, severe inflammation. Detailed pathological assessment was performed as described previously [35].

### ROS detection in vivo

Renal ROS levels were estimated using a chemoluminescence assay for superoxide anion, and the results were presented as reactive luminescence units (RLU) per 15 min per milligram dry weight (i.e., RLU/15 min/mg dry weight) as described previously [36]. The levels of ROS in serum and urine were tested following the instructions on the hormonal ROS assay kit (GeneMed, USA). According to reports, we used ROS Fluorescent Probe-dihydroethidium (DHE; Invitrogen, Carlsbad, CA, USA) to detect the level of ROS in the kidney.

### Measurement of cellular glutathione peroxidase activity in the kidney

Renal cortex was lysed using RIPA lysis solution. The glutathione peroxidase (GPx) activity in renal cortex lysates was measured using a commercial GPx assay kit (Cayman, MI, USA) according to the manufacturer’s protocols.

### Plasma cytokine ELISA

Total urinary protein was determined using a Mouse Albumin ELISA Quantitation Set (Bethyl Laboratories) according to the manufacturer’s instructions, and the urine was applied at dilutions of 1:100. Anti-IgG and anti-dsDNA IgG were analyzed using a mouse anti-IgG and anti-dsDNA IgG Kit (Bethyl Laboratories) according to the manufacturer’s instructions, and the sera were applied at dilutions of 1:100,000 and 1:500,000. Cytokines were analyzed using a mouse IL-18 and IL-1β ELISA Kit (R&D Systems) according to the manufacturer’s instructions. Absorbance at 450 nm was determined using an ELx-800 Universal Microplate Reader (BioTek).

### Precipitation of soluble proteins in supernatants

Soluble protein in culture supernatants was precipitated as previously described [37]. The precipitated proteins were dissolved in equal volume of 1× SDS-PAGE sample loading buffer and subjected to the western blot analysis of secreted mature IL-1β and caspase-1 p20.

### Apoptosis assay

MDSC apoptosis was measured by flow cytometry using an Annexin V-FITC apoptosis detection kit. Briefly, MDSCs were treated with different concentrations. Then MDSCs were stained with Annexin V-FITC and propidium iodide (PI) at room temperature for 15 min in the dark. After removing the unbound Annexin V-FITC and PI by centrifugation, cells were resuspended in 500 μl binding buffer. The apoptotic cells were analyzed by a FACS flow cytometer (Becton Dickinson, CA, USA).

### Flow cytometry analysis

BM cells were isolated as described previously from mice by flushing femurs and tibiae. Kidneys were prepared to single-cell suspensions with collagenase type D (1 mg/ml) and DAase I (0.1 mg/ml) in HBSS at 37 °C for 30 min, and then the red cells from the kidneys were lysed. For cell surface marker staining, splenocytes, BM cells, PBMCs, and kidney cells from mice were prepared as single-cell suspensions. The cell suspensions were filtered through 70-μm cell strainers, and the lymphocytes were collected by centrifugation at 300 g for 5 min at 4 °C. After washing, the cells were immediately prepared for flow cytometry. For the detection of mouse MDSC subsets, cells were pre-incubated with FITC-conjugated anti-mouse CD11b mAb and APC-conjugated anti-mouse Gr-1 mAb and then cells were stained for 30 min at 4 °C in the dark. For the detection of mouse macrophages, cells were pre-incubated with FITC-conjugated anti-mouse CD11b and APC-conjugated anti-mouse F4/80 mAb and then cells were stained for 30 min at 4 °C in the dark. Cells were stained with FITC-conjugated anti-mouse CD4 mAb and APC-conjugated anti-mouse CD3 mAb for detection of T cell infiltration in the spleen. Cells were stained with APC-conjugated anti-mouse CD69 mAb and FITC-conjugated anti-mouse B220 mAb or FITC-conjugated anti-mouse CD4 mAb for detection of activated B cells and T cells respectively. In addition, cells were stained with APC-conjugated anti-mouse CD11C mAb and FITC-conjugated anti-mouse MHCII mAb for dendritic cell detection.

### RNA extraction and quantitative real-time PCR

Total RNA was isolated using Trizol Reagent according to the manufacturer’s instructions. Real-time PCR assay was performed using SYBR green dye on a Step One sequence detection system (Applied Biosystems, Waltham, MA, USA). Relative abundance of genes was calculated using the 2^−ΔΔCT^ formula, and GAPDH was an internal control. The primers are listed in Additional file 1: Table S1.

### Western blot analysis

Proteins were extracted by standard techniques [38]. In short, total proteins were separated by SDS-PAGE and electro-transferred to PVDF membranes. Then, membranes were blocked in 5% BSA dissolved in TBST (50 mM Tris/HCL, pH 7.6, 150 mM NaCl, and 0.1% Tween-20) for 2 h at room temperature and incubated with indicated primary antibody overnight at 4 °C, followed by incubation with appropriate HRP-linked secondary antibody 2 h at room temperature. Protein bands were visualized using ECL Plus Western blotting detection reagents (Millipore, Bedford, MA, USA). The blot images were captured by a FluorChem8000 imaging system (AlphaInnotech, San Leandro, CA, USA). The gray values were analyzed by ImageJ gel analysis software.

### Immunofluorescence staining

Frozen sections of kidneys were stained with anti-Gr-1 (BD Pharmingen, USA) and anti-NLRP3 (CST, USA) followed by treatment with HRP-conjugated anti-rat IgG (Dako), and were visualized using diaminobenzidine (DAB) and hematoxylin as counterstaining. Frozen sections of kidneys were treated with anti-WT-1 (Merck Millipore, Bedford, MA, USA) followed by treatment with Alexa Fluor 488-conjugated goat anti-mouse IgG (Invitrogen, Carlsbad, CA). MDSCs were collected and stained with anti-Nrf2 (Abcam, USA) followed by treatment with HRP-conjugated anti-rat IgG (Dako). Fluorescence images were captured by a laser scanning confocal microscope (FV3000, Olympus Corporation, Japan).

### Statistical analysis

Results were expressed as mean ± standard error of the mean (SEM) of three independent experiments, and each experiment included triplicate sets. Data were statistically evaluated by one-way analysis of variance followed by the Dunnett’s test between control group and multiple dose groups. *P* ≤ 0.05 was considered of statistically significant difference.

## Results

### Baicalein ameliorates the disease activity in pristane-induced lupus nephritis

**Fig. 1:**
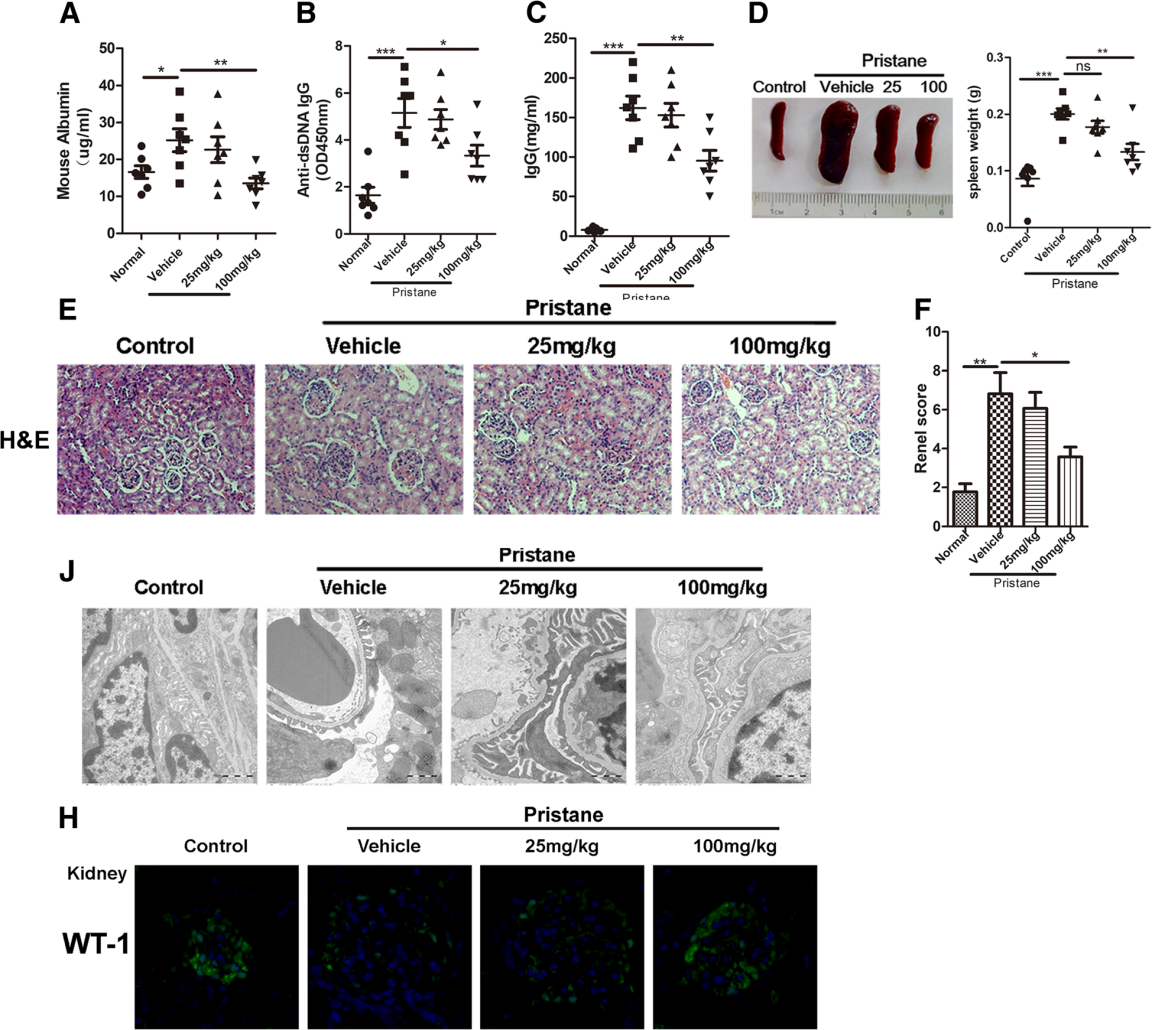
Baicalein ameliorates the disease activity in pristane-induced lupus nephritis. BALB/c WT mice (*n* = 7/group) were given a single injection of 0.5 ml pristane and kept for 5 months. Then mice were randomly divided into three groups: vehicle, 25 mg/kg baicalein, 100 mg/kg baicalein, and kept for another 2 months. **a** Proteinuria in each group was determined using a Mouse Albumin ELISA Quantitation Set. **b**, **c** The levels of serum anti-dsDNA IgG and IgG were detected by ELISA. **d** Representative photographs of the spleen from each group (left) and the spleen weight of each group (right). **e** Kidney histopathological evaluation by H&E staining. **f** Glomerulonephritis score of each group. **j** Assessment of podocyte foot processes by transmission electron microscopy. Original magnification × 6000. **h** Representative confocal images of kidneys from each group stained with DAPI (blue) and anti-WT-1 (green) showed podocyte injury. BA represents baicalein. Data represent the mean scores ± SEM. *P ≤ 0.05, **P ≤ 0.01, ***P ≤ 0.001. *n* = 7 animals per group

To determine the therapeutic effects of baicalein on pristane-induced LN, BALB/c mice were injected with 0.5 ml pristane (*n* = 7/group). After 5 months, lupus mice were treated with or without 25 mg/kg or 100 mg/kg baicalein, respectively, for another 2 months. As shown in Fig. 1, baicalein significantly lowered the level of urinary protein in lupus mice (Fig. 1a). Compared with the vehicle-treated mice, 100 mg/kg baicalein decreased the levels of anti-dsDNA IgG (Fig. 1b) and IgG (Fig. 1c). Baicalein gradually relieved splenomegaly (Fig. 1d) and lung inflammation (Additional file 1: Figure S1A) in lupus mice. Moreover, histological assessment showed that baicalein remitted severe inflammatory infiltrates and bone erosion in the tarsal joints (Additional file 1: Figure S1B). Baicalein also mitigated glomerulonephritis and infiltration of lymphocytes (Fig. 1e–f). Transmission electron microscopy showed that baicalein notably attenuated foot process effacement (Fig. 1j). In addition, expression of WT1, a marker of podocyte, was significantly decreased in vehicle-treated lupus mice, while baicalein gradually enhanced WT1 expression (Fig. 1h). These data demonstrated that baicalein ameliorated the disease activity in pristane-induced LN.

### Baicalein blocks inflammation and oxidative stress in pristane-induced lupus nephritis

**Fig. 2:**
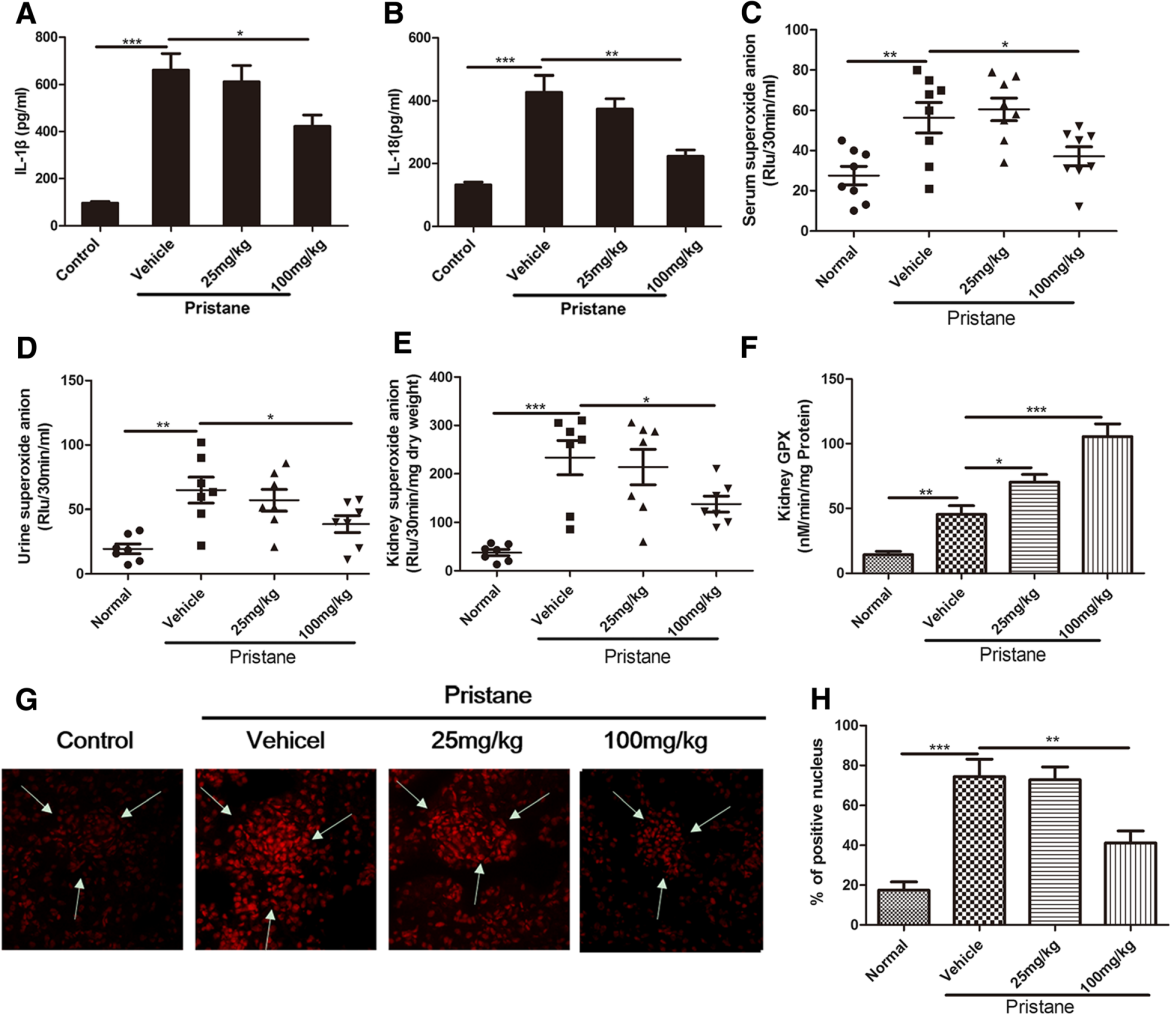
Baicalein blocks inflammation and oxidative stress in pristane-induced lupus. Mice were treated as described in Fig. 1. **a**, **b** The levels of IL-1β and IL-18 in the serum were detected by ELISA. **c** Superoxide anion levels in the serum, **d** urine, and **e** kidney were detected. **f** GPx activity in the kidney. **g** Kidney ROS production was demonstrated by dihydroethidium (DHE) labeling. The arrows indicated the glomeruli. Original magnification, × 200. **h** The scoring of the percentage of positive nuclei is shown in the right panel. BA represents baicalein. Data represent the mean scores ± SEM. *P ≤ 0.05, **P ≤ 0.01, ***P ≤ 0.001. *n* = 7 animals per group

To investigate whether amelioration of baicalein on pristane-induced LN was related to the inhibition of inflammation and oxidative stress, some associated indexes were examined. The results showed that the serum levels of IL-1β and IL-18 remarkably increased in lupus mice, while baicalein significantly inhibited the production of these cytokines (Fig. 2a, b). Baicalein also reduced the serum levels of IFN-α, IL-17A, and IL-6 in lupus mice (Additional file 1: Figure S2B-D), but the effect of baicalein on the serum level of IFN-γ was slight (Additional file 1: Figure SA). Moreover, superoxide anion levels significantly increased in serum (Fig. 2c), urine (Fig. 2d), and kidney (Fig. 2e) of lupus mice, while baicalein decreased the superoxide anion levels to normal. Of note, baicalein markedly induced the activity of glutathione peroxidase (GPx) (Fig. 2f), which is one of the phase II enzymes downstream of the antioxidant Nrf2 pathway [39, 40], in the kidneys. Baicalein also lowered DHE fluorescence in kidney of lupus mice, indicating ROS production was decreased in renal tissue (Fig. 2g, h). These results suggested that the inhibition effects of baicalein on inflammation and oxidative stress might be related to alleviation of pristane-induced LN.

### Anti-inflammatory/oxidative abilities of baicalein depend on the upregulated Nrf2/HO-1 and downregulated NLRP3/NF-κB in pristane-induced lupus nephritis

**Fig. 3:**
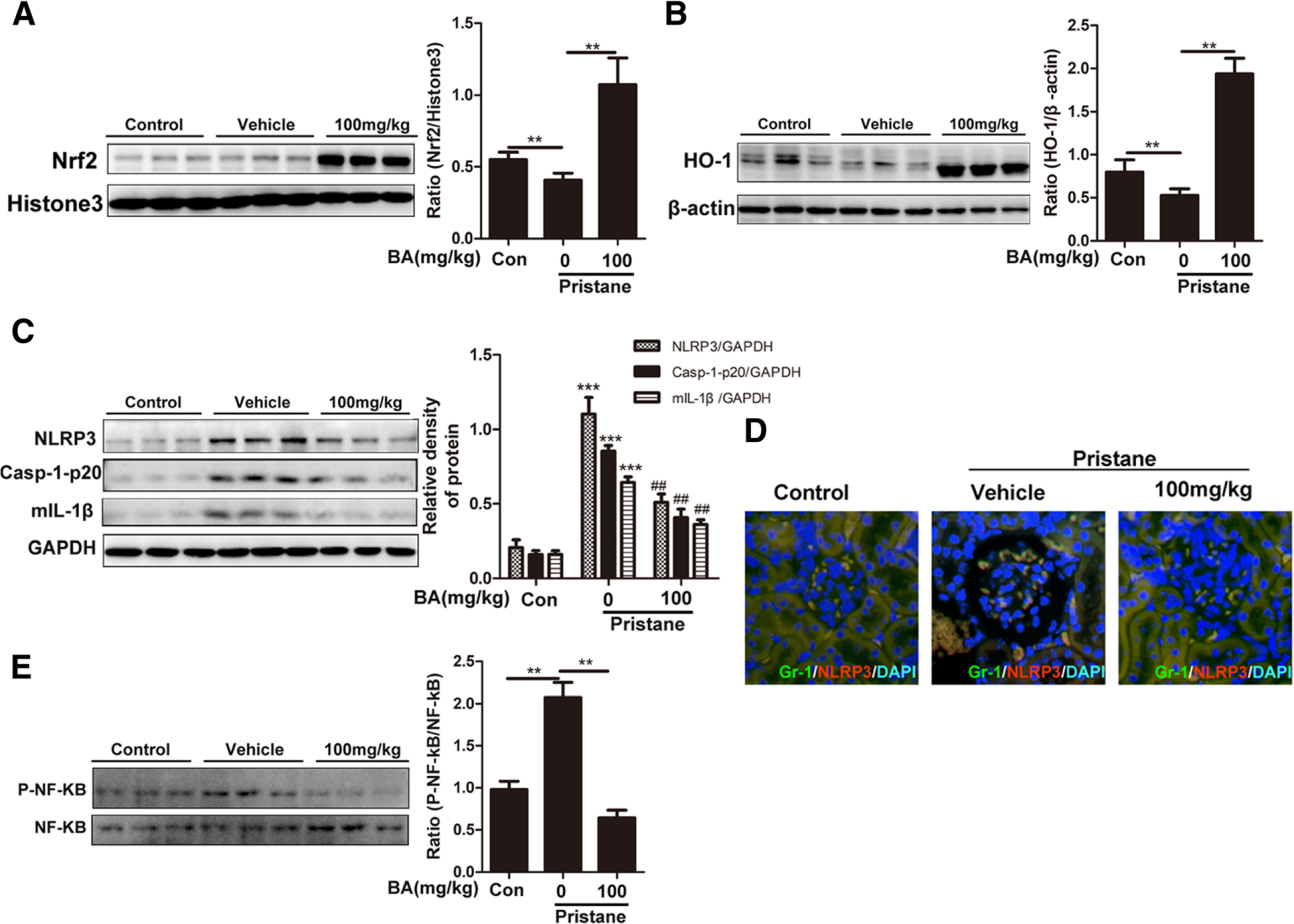
Anti-inflammatory/oxidative abilities of baicalein depends on the upregulated Nrf2/HO-1 and downregulated NLRP3/NF-κB in pristane-induced lupus nephritis. Mice were treated as described in Fig. 1. **a** Representative western blots for nuclear levels of Nrf2, with Histone3 as the loading control. **b** Representative western blots for nuclear levels of HO-1, with β-actin as the loading control. **c** Representative western blots for cytosolic levels of NLRP3, Casp-1-p20, and mIL-1β in the kidney, with β-actin as the loading control. **d** Immunofluorescence of glomerular GR-1, represents MDSCs, and NLRP3 in each group. **e** Representative western blots for nuclear levels of P-NF-κB, with NF-ΚB as the loading control. BA represents baicalein. Data represent the mean scores ± SEM. *P ≤ 0.05, **P ≤ 0.01, ***P ≤ 0.001. ^#^*P* < 0.05 and ^##^*P* < 0.01. The 100 mg/kg baicalein treatment group vs the pristane group. *n* = 7 animals per group

Accumulated evidence revealed that the protective effects of Nrf2 against SLE in some cellular and mouse models [17, 41] and the activation of the NLRP3 inflammasome and NF-κB pathway contributed to the development of LN [3, 2, 17, 42, 43]. Here we determined the changes of Nrf2, NLRP3, or NF-κB in pristane-induced lupus. As expected, baicalein evoked a significant increase in Nrf2 expression and its target enzymes HO-1 in kidney tissue of lupus mice (Fig. 3a, b). In contrast, baicalein inhibited the protein expression of NLRP3 inflammasome (Fig. 3c). The immunofluorescent intensity of NLRP3 inflammasome in MDSCs was fainter in the baicalein treatment group compared with the model group (Fig. 3d). Moreover, to determine the blockage of baicalein on inflammasome activation, the post-translational processing of procaspase-1 (Casp-1-p20) and the mature form of the pro-inflammatory cytokine IL-1β (mIL-1β) were measured. Western blot analysis showed that baicalein decreased mature caspase-1 p20 subunit and IL-1β in kidneys of lupus mice (Fig. 3c). In addition, baicalein also markedly reduced the level of phosphorylated p65 compared with the vehicle group (Fig. 3e). These results suggested that anti-inflammatory and anti-oxidative abilities of baicalein might be dependent on the upregulation of the Nrf2/HO-1 signaling and downregulation of NLRP3/NF-κB activation.

### Suppression of baicalein on MDSC inflammatory/oxidative abilities is involved in the amelioration of pristane-induced lupus nephritis

**Fig. 4:**
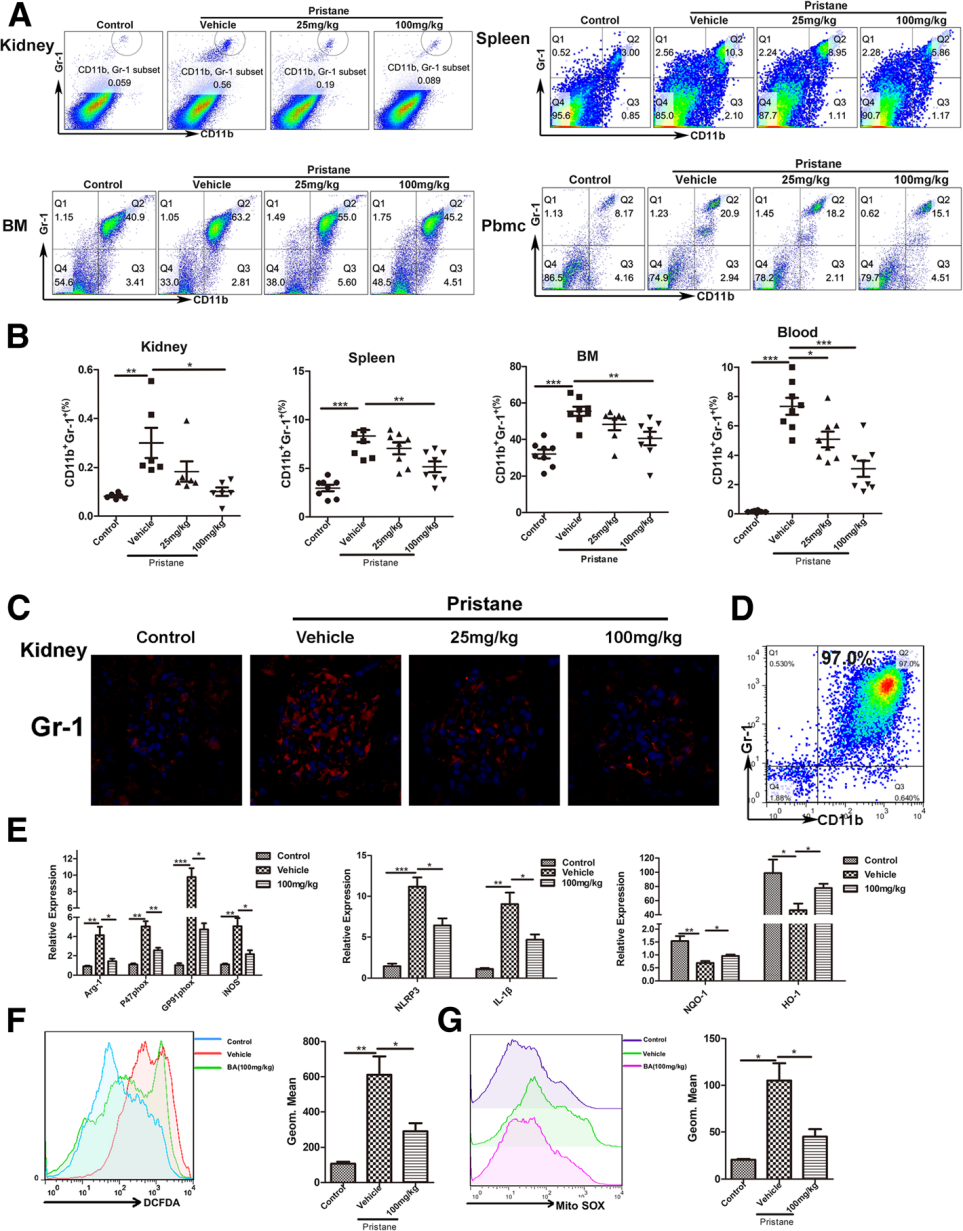
Suppression of baicalein on MDSC inflammatory/oxidative abilities is involved in amelioration of pristane-induced lupus nephritis. Mice were treated as described in Fig. 1. **a** The frequency of MDSCs were determined by FACS in renal, splenic, BM, and Pbmc. **b** The statistical results of the frequency of MDSCs. **c** Immunofluorescence of glomerular GR-1 represents MDSCs. **d** The purity of sorted MDSCs from spleen was determined via flow cytometry. **e** Relative expression of Arg-1, P47 ^phox^, GP91 ^phox^, iNOS, NQO-1, HO-1, NLRP3, and IL-1β mRNA in MDSCs purified from the spleen were measured by QPCR. **f**, **g** ROS and MitoSOX production in MDSCs purified from the spleen was analyzed by flow cytometry. BA represents baicalein. Data represent the mean scores ± SEM. *P ≤ 0.05, **P ≤ 0.01, ***P ≤ 0.001. *n* = 7 animals per group

Although MDSCs’ role in the development of lupus was controversial, we previously found that MDSCs were elevated in both SLE patients and MRL/lpr or IMQ-lupus-prone mice [11–13]. We first confirmed changes of MDSCs in pristane-induced lupus mice. The results showed that the number of MDSCs increased in lupus mice, while baicalein (100 mg/kg) significantly decreased the percentages of MDSCs in the kidney, spleen, bone marrow (BM), and Pbmc (Fig. 4a, b). Of note, baicalein also decreased the intensity of immunofluorescence staining for Gr-1^+^ in the kidney of lupus mice (Fig. 4c). In addition, we found that baicalein reduced the total T cells and CD4+ T cell infiltrations in the spleen compared with the model group (Additional file 1: Figure S3A). Meanwhile, we suggested that baicalein reduced the activated T cells and had a slight effect on the activated B cells in the spleen compared with the vehicle group (Additional file 1: Figure S3B-C). Baicalein inhibited the macrophage cells and mature dendritic cell infiltrations in the spleen compared with the vehicle group (Additional file 1: Figure S3D-E). Meanwhile, the percentage of macrophage cells in the kidney of the baicalein group lowered than that of the vehicle group (Additional file 1: Figure S3F).

To explore the effect of baicalein on oxidative stress and inflammation in MDSCs isolated from the spleen of each group, we used quantitative real-time PCR to measure indexes of oxidative stress, including Arg-1, P47 ^phox^, GP91 ^phox^, and iNOS, the target gene of the antioxidant Nrf2 pathway, including NQO-1 and HO-1, and the indexes of NLRP3 inflammasome, including NLRP3 and IL-1β. The purity of sorted MDSCs from spleen was determined via flow cytometry (Fig. 4d). As expected, baicalein significantly reduced relative expression of the above genes besides elevating genes of the antioxidant Nrf2 pathway in MDSCs isolated from the spleen (Fig. 4e). It also decreased the levels of ROS and MitoSOX (mitochondrial superoxide indicator for live-cell imaging) in MDSCs purified from the spleen (Fig. 4f). These results indicated that the downregulatory effect of baicalein on oxidative stress and inflammation in MDSCs might be necessary for ameliorating pristane-induced mouse LN.

### Both NLRP3/NF-κB inhibition and Nrf2 promotion are verified in effect of baicalein on MDSCs in vitro

**Fig. 5:**
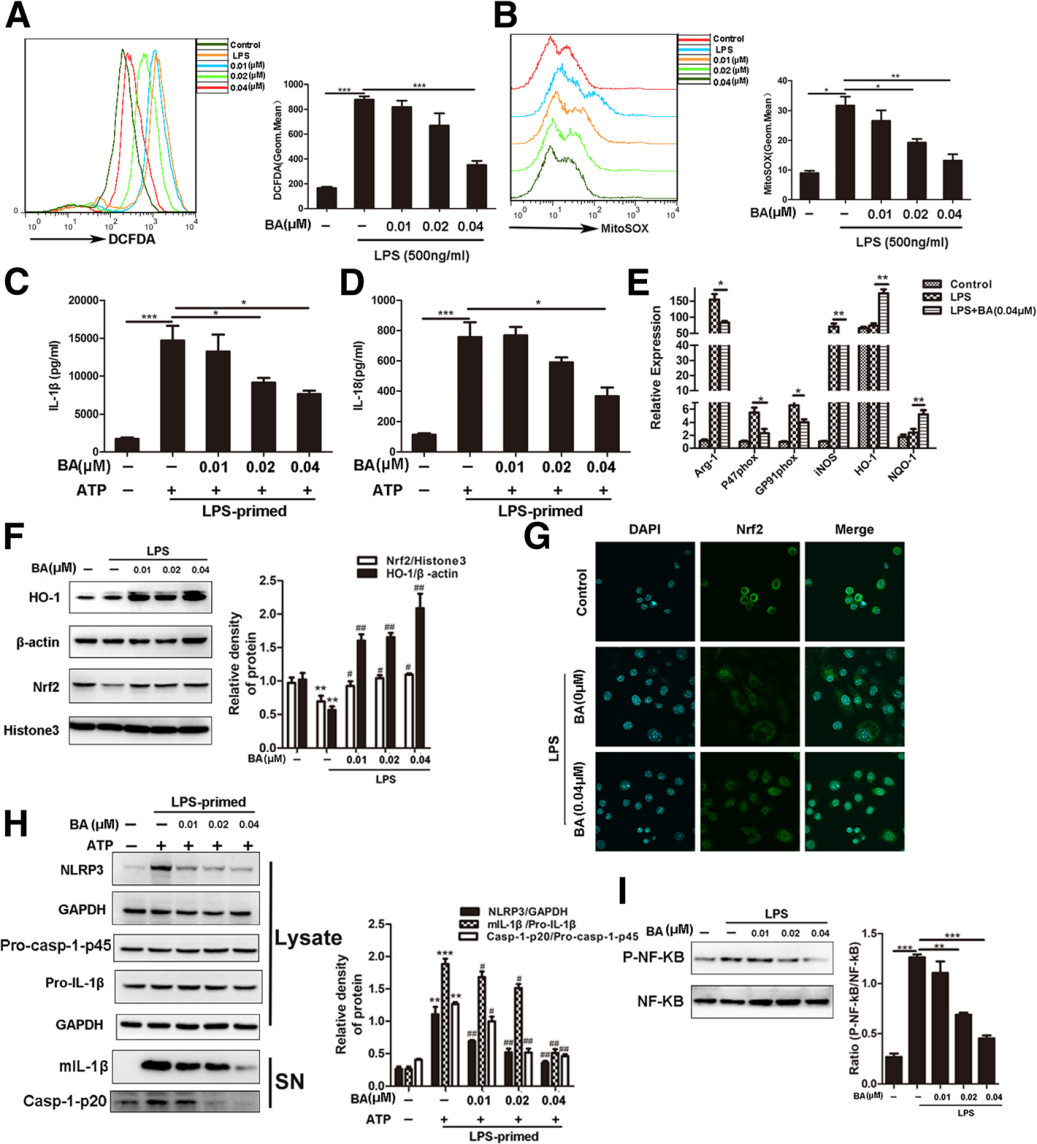
Both NLRP3/NF-κB inhibition and Nrf2 promotion are verified in effect of baicalein on MDSCs in vitro. MDSCs were treated with or without baicalein 3 h after being primed for 1 h for different concentrations (0.01 μM, 0.02 μM, 0.04 μM), then treated with or without ATP (5 mM) for another 30 min. **a**, **b** ROS and MitoSOX production in MDSCs was analyzed by flow cytometry. **c**, **d** IL-1β and IL-18 secretion was detected by ELISA. **e** Relative expression of Arg-1, P47 ^phox^, GP91 ^phox^, iNOS, NQO-1, and HO-1 mRNA in MDSCs were measured by QPCR. **f** Nuclear levels of Nrf2 and the protein expression of HO-1 were determined by western blot. **g** Immunofluorescence of Nrf2. **h** Western blotting was used to assess the expression levels of NLRP3, Pro-casp-1, and Pro-IL-1β proteins in the cell lysates, and Casp-1-p20 and mIL-1β in culture supernatants, respectively; GAPDH was used as a loading control for cell lysates. **i** P-NF-κB and NF-κB were determined by western blot. BA represents baicalein. Data represent the mean scores ± SEM of triplicate experiments. *P ≤ 0.05, **P ≤ 0.01, ***P ≤ 0.001, ^#^*P* < 0.05, and ^##^*P* < 0.01 vs LPS with the ATP group

To verify that baicalein protected MDSCs against oxidative stress and inflammation, Nrf2 and NLRP3/NF-κB were detected. MDSCs were generated from BM cells and then stimulated with or without baicalein 3 h after LPS (500 ng/ml) primed 1 h in different concentrations (0.01 μM, 0.02 μM, 0.04 μM), followed by treatment with or without ATP (5 mM) for another 30 min. The effect of baicalein on MDSC viability was measured by staining with Annexin V and the CCK8 assay. The results showed that baicalein had no effect on MDSC viability below 0.06 μM (Additional file 1: Figure S4). Moreover, baicalein decreased the accumulation of intracellular ROS and MitoSOX in LPS-primed MDSCs (Fig. 5a, b). It also suppressed ATP-induced secretion of mature IL-1β and mature IL-18 in MDSC supernatants (Fig. 5c, d). Moreover, it reduced mRNA expression of oxidative stress indexes including Arg-1, P47 ^phox^, GP91 ^phox^, and iNOS but elevated the expression of Nrf2 and HO-1 in LPS-primed MDSCs (Fig. 5e–g). The expression of NLRP3 protein in MDSCs was highly induced by LPS, whereas pro-IL-1β and procaspase-1 were constitutively expressed in MDSCs irrespective of LPS priming. Upon ATP treatment, both active casp-1-p20 and mature IL-1β were released into the culture supernatants. Baicalein robustly suppressed the expression of NLRP3 and the release of activated casp-1-p20 and mIL-1β into the culture supernatant (Fig. 5h). It also markedly reduced the level of phosphorylated p65 in LPS-primed MDSCs (Fig. 5i). These results indicated that the inhibition of NLRP3/NF-κB and promotion of Nrf2 might be critical in the ameliorating effect of baicalein on oxidative stress and inflammation in MDSCs.

### Baicalein anti-inflammatory/oxidative abilities are mainly attributed to the promotion of Nrf2 signaling in MDSCs

**Fig. 6:**
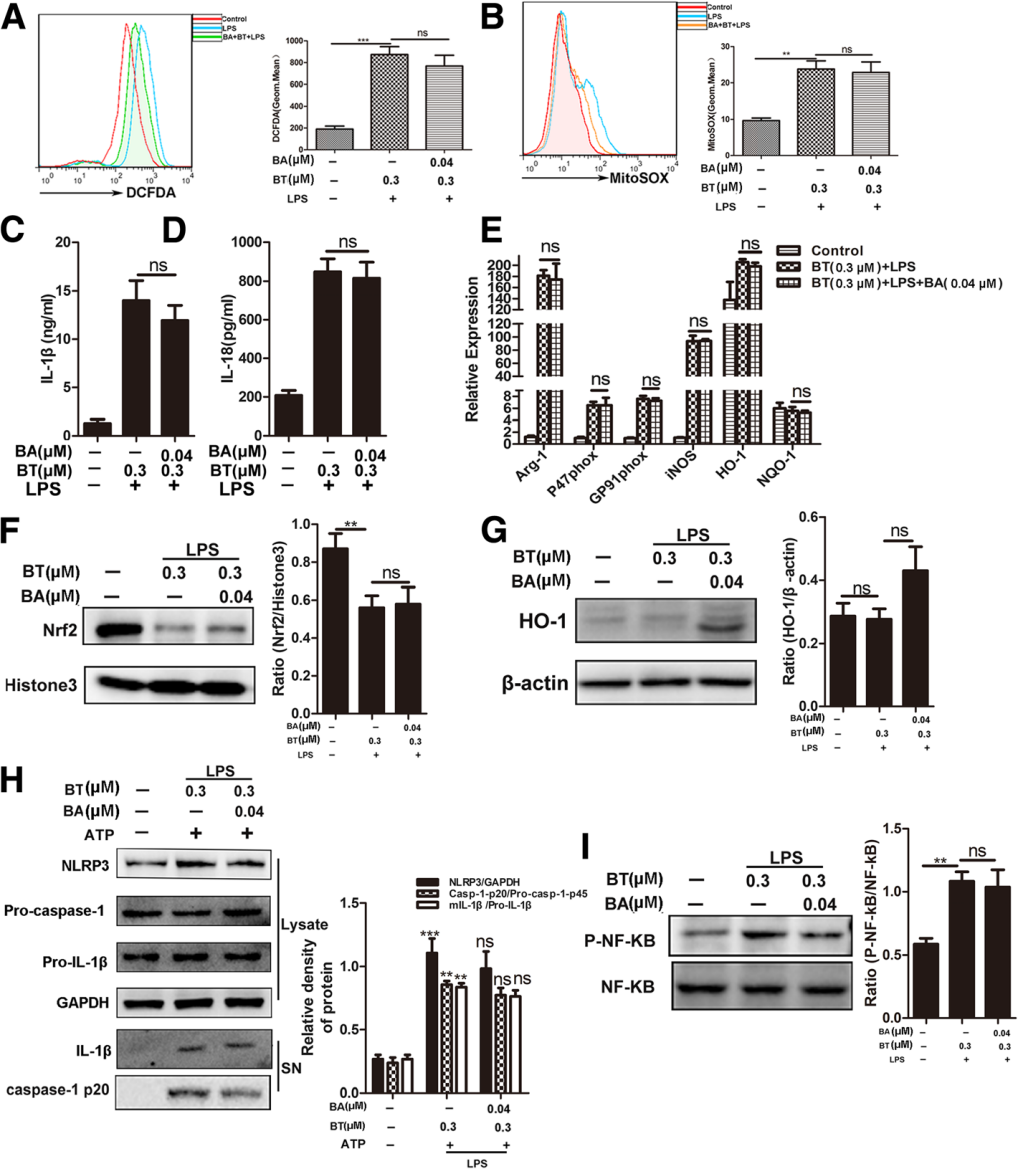
Baicalein anti-inflammatory/oxidative abilities are mainly attributed to the promotion of Nrf2 signaling in MDSCs. MDSCs were treated with or without BT (0.3 μM) for 1 h, a specific inhibitor of Nrf2, and then with or without baicalein (0.04 μM) for 1 h prior to incubation with LPS (500 ng/ml) for another 5 h, followed by incubation with or without ATP (5 mM) for 30 min. **a**, **b** ROS and MitoSOX production in MDSCs was analyzed by flow cytometry.**c**, **d** IL-1β and IL-18 secretion was detected by ELISA. **e** Relative expression of Arg-1, P47 ^phox^, GP91 ^phox^, iNOS, NQO-1, and HO-1 mRNA in MDSCs were measured by QPCR. **f**, **g** Nuclear levels of Nrf2 and the protein expression of HO-1 were determined by western blot. **h** Western blotting was used to assess the expression levels of NLRP3, Pro-casp-1, and Pro-IL-1β proteins in the cell lysates, and Casp-1-p20 and mIL-1β in culture supernatants, respectively; GAPDH was used as a loading control for cell lysates. **i** P-NF-κB and NF-κB were determined by western blot. BA represents baicalein. Data represent the mean scores ± SEM of triplicate experiments. *P ≤ 0.05, **P ≤ 0.01, ***P ≤ 0.001

To determine the main signaling for baicalein to protect MDSCs against oxidative stress and inflammation, we treated MDSCs with or without brusatol (0.3 μM), a specific inhibitor of Nrf2, for 1 h and then treated with or without baicalein (0.04 μM) for 1 h prior to incubation and with or without LPS (500 ng/ml) for another 5 h, followed by ATP (5 mM) for 30 min. The results showed that Nrf2 inhibition not only impeded the attenuating effect of baicalein on intracellular ROS and MitoSOX levels (Fig. 6a, b) but also substantially blocked the secretion of mature IL-1β and IL-18 (Fig. 6c, d) in MDSCs. Baicalein also could not significantly trigger the gene expression of Arg-1, P47 ^phox^, GP91 ^phox^, iNOS, NQO-1, and HO-1 in LPS-primed MDSCs with Nrf2 inhibition (Fig. 6e). Certainly, Nrf2 inhibition suppressed the protein expression levels of Nrf2 and HO-1 (Fig. 6f, g) and NLRP3 inflammasome activation (Fig. 6h) in MDSCs. In addition, Nrf2 inhibition also influenced the ability of baicalein to downregulate the phosphorylation of NF-κB p65 in LPS-primed MDSCs (Fig. 6i). Altogether, these data indicated that the protective effect of baicalein on MDSCs against oxidative stress and inflammation was attributed to the inhibition of the NLRP3/NF-κB pathway in a Nrf2-dependent pattern.

## Discussion

Recent studies showed that the changes in MDSCs were associated with the progression of LN [1, 7–9, 11, 44, 45]. However, the exact role of MDSCs in the pathogenesis of LN remains unclear. Oxidative stress means a perturbed redox signaling and is reported to have an interactive relationship with inflammation. Oxidative stress is intimately involved in multiple diseases, including LN [46, 47]. Baicalein is a member of the flavonoid family, which owns antioxidant and anti-inflammatory activities [28–30]. It also enhanced the expression of Nrf2 pathway to reduce oxidant stress in colistin-induced nephrotoxicity mice [28] and inhibited the activation of NLRP3 inflammasome in TNBS-induced colitis [48]. Our results in this study unveiled that baicalein could ameliorate LN by reducing the number of MDSCs and blocking the oxidative stress and inflammation in MDSCs.

We found that baicalein suppressed the progress of lupus nephritis, as illustrated by improved indexes of pathology, including decreased albuminuria, improved renal function, inflammatory cell infiltration, and podocyte injuries (Fig. 1a, e–h). It also decreased IL-1β and IL-18 production in serum (Fig. 2a, b) and ROS levels in serum, urine, and kidney tissue as well as levels of superoxide and GPx in the kidney (Fig. 2c–h). It is widely accepted that the transcription factor Nrf2 effectively reduces ROS levels [45, 47]. Nevertheless, persistent and uncontrolled inflammation is also intimately associated with oxidative stress and implicated in LN. Among the various inflammatory responses, the NF-κB and NLRP3 pathways are reported to participate in the LN progress [18, 41, 42, 48, 49]. In the present study, baicalein also increased the expression levels of nuclear Nrf2 and its target antioxidant enzymes including HO-1 in kidney tissue. Meanwhile, the present study verified the inhibitory effects of baicalein on both the NF-κB and NLRP3 pathways in kidney tissue from each group (Fig. 3).

Some studies suggested that MDSCs were pro-inflammatory to promote the disease progress in chronic inflammation conditions [10–13, 49]. Therefore, we presumed that the reduction of percentage and oxidative stress in MDSCs might alleviate LN. We founded that baicalein reduced the percentage of MDSCs in lupus mice (Fig. 4a, b). In addition, we suggested that baicalein reduced the infiltrations of total T cells and CD4+ T cells in the spleen compared with the vehicle group (Additional file 1: Figure S3A). It reduced the activated T cells and had a slight effect on activated B cells in the spleen compared with the vehicle group (Additional file 1: Figure S3B-C). Some research have demonstrated that baicalein could inhibit the proliferation of T cells and B cells in the spleen [50–53], but whether baicalein had a regulatory role on MDSCs had not been reported. In addition, baicalein inhibited the macrophage cells and mature dendritic cell infiltrations in the spleen compared with the vehicle group (Additional file 1: Figure S3D-E). Meanwhile, the percentage of macrophage cells in the kidney of the baicalein group lowered than that of the vehicle group (Additional file 1: Figure S3F).

Macrophage cells and dendritic cells were believed to contribute to the pathogenesis of SLE [54]. Some studies reported that MDSCs could accumulate in many organs in chronic inflammation conditions. The accumulation of MDSCs had the potential of differentiation to macrophage cells and dendritic cells [7]. We have shown that baicalein reduced the percentage of MDSCs in the spleen and kidney compared with the vehicle group in Fig. 4a. Therefore, baicalein downregulated the expansions of macrophage cells and dendritic cells in pristane-induced lupus mice, which might be attributed to the dropped percentage of MDSCs in baicalein treatment. These data suggested that the alleviation of baicalein on LN might be linked to its ability to regulate the changes in MDSCs.

We further found that baicalein could protect MDSCs from oxidative stress and inflammation in vitro. Baicalein decreased the levels of ROS and MitoSOX in MDSCs derived from BM cells under conditions of LPS-induced oxidative stress and inflammation (Fig. 5a, b). As expected, we discovered that baicalein significantly reduced ATP-induced secretion of mature IL-1β and mature IL-18 in cell supernatant from MDSCs (Fig. 5c, d). Baicalein also increased the protein expression of Nrf2 and HO-1 in MDSCs (Fig. 5f, g). Furthermore, we observed that baicalein blocked the protein expression levels of NLRP3 inflammasome and phosphorylated NF-κB p65 increased by the combination of LPS and ATP or by just LPS stimulation in MDSCs (Fig. 5h, i). These findings suggested that baicalein might protect against LN through the suppression of oxidative stress and inflammation in MDSCs, which associated with the inhibition of NLRP3/NF-κB activation and promotion of the expression of the Nrf2 signal.

The relationship between Nrf2 and pro-inflammatory pathways is still controversial [14, 28, 46, 55–58]. To investigate the crosstalk between Nrf2 and pro-inflammatory pathways, pretreatment of brusatol (BT, a specific inhibitor of Nrf2) abolished the protective effects of baicalein on oxidative stress and inflammation in LPS-primed MDSCs (Fig. 6a–d). Surprisingly, the promotion effects of baicalein on Nrf2/HO-1 antioxidant pathway were also downregulated in LPS-primed MDSCs pretreated with BT (Fig. 6e–g). As expected, baicalein failed to inhibit the increase in NLRP3 inflammasome and phosphorylated NF-κB p65 expression in LPS-primed MDSCs pretreated with BT (Fig. 6h, i).These results suggested that baicalein suppressed oxidative stress and inflammation in LPS-primed MDSCs through the inhibition of NLRP3/ NF-κB in an Nrf2-dependent way.

**Fig. 7:**
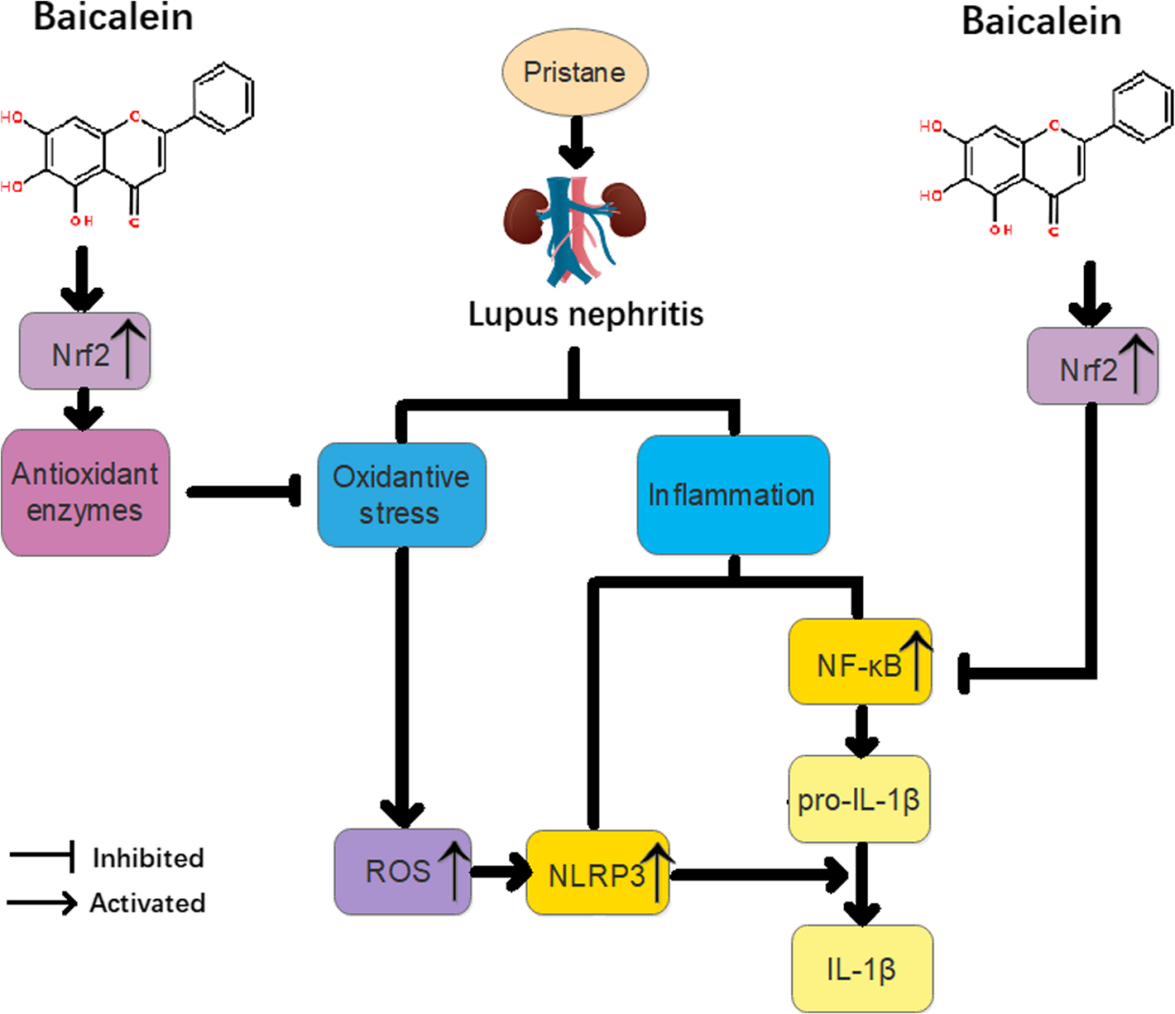
Scheme summarizing the protective effect of baicalein on pristane-induced lupus nephritis (LN) and underlying mechanisms. Baicalein treatment significantly alleviated pristane-induced LN via the restriction of oxidative and inflammatory injury, which was associated with activation of Nrf2/HO-1 response element signaling and inhibition of NLRP3 and NF-κB pathways

In summary, we found that baicalein reduced proteinuria and attenuated renal function impairment and renal histopathology in lupus mice. Baicalein decreased activation of NLRP3 inflammasome and levels of ROS or NF-kB phosphorylation, but it enhanced Nrf2 activation. Baicalein inhibited the expansion of MDSCs and improved the function of MDSCs in lupus mice. Baicalein exhibited cytoprotective effects coincided with the induction of Nrf2/HO-1 signaling and the suppression of the NLRP3 inflammasome in MDSCs (Fig. 7).

## Conclusion

Baicalein alleviates symptoms of pristane-induced LN and suggests that the alleviation may be attributed to inhibition of MDSC expansion and regulation of the balance of Nrf2/HO-1 signal and NLRP3 expression in MDSCs.

## Additional file

Additional file 1: **Figure S1.** Baicalein attenuates inflammation of lung and joint in pristane-induced lupus mice. BALB/c WT mice (*n* = 7/group) were given a single injection of 0.5 ml pristane and kept for 5 months. Then mice were randomly divided into three groups: vehicle, 25 mg/kg baicalein, 100 mg/kg baicalein and kept for another 2 months. (A) Lung sections from each groups showed histologic differences. (B) Representative histological sections of tarsal hind paw joints showing normal appearance and severe inflammatory infiltration and bone loss. Data represent the mean scores ± SEM. *P ≤ 0.05, **P ≤ 0.01, ***P ≤ 0.001. *n* = 7 animals per group. **Figure S2.** Baicalein attenuates the serum level of pro-inflammatory cytokines in lupus mice. (A) The level of IFN-γ in serum. (B) The level of IFN-α in serum. (C) The level of IL-17A in serum. (D) The level of IL-6 in serum. Data represent the mean scores ± SEM. *P ≤ 0.05, **P ≤ 0.01, ***P ≤ 0.001. *n* = 7 animals per group. **Figure S3.** Baicalein reduces the expansion of inflammatory cell in pristane-induced lupus mice. (A) The percentage of total T cells in spleen. (B) The percentage of activated T cells in spleen. (C) The percentage of activated B cells in spleen. (D) The percentage of macrophage cells in spleen. (E) The percentage of mature dendritic cells in spleen. (F) The percentage of macrophage cells in kidney. Data represent the mean scores ± SEM. *P ≤ 0.05, **P ≤ 0.01, ***P ≤ 0.001. *n* = 7 animals per group. **Figure S4.** The effect of baicalein on MDSCs apoptosis. (A) BM cells from 6-8w female mice were cultured for 4 days with GM-CSF (40 ng/ml) and IL-6 (40 ng/ml), the proportions of CD11b^+^Gr-1^+^ MDSCs were analyzed by flow cytometry. (B) The statistical results of the frequency of MDSCs. (C) MDSCs were treated with BA (0.01 μM, 0.02 μM, 0.04 μM, 0.06 μM) for 24 h and the apoptosis cells were detected with Annexin V by flow cytometry. (D) The cell viability was determined by a CCK8 assay. (E) The cell cycle was determined by flow cytometry. Data represent the mean scores ± SEM of triplicate experiments. *P ≤ 0.05, **P ≤ 0.01, ***P ≤ 0.001. **Table S1.** Primers of mouse gene used for real-time RT-PCR. (DOCX 4600 kb)

## Abbreviations

ATP: Adenosine triphosphate
BA: Baicalein
BT: Brusatol
DMSO: Dimethyl sulfoxide
ds: Double strand
ELISA: Enzyme-linked immunosorbent assay
FITC: Fluorescein isothiocyanate
GAPDH: Glyceraldehyde 3-phosphate dehydrogenase
GPx: Glutathione peroxidase
HO-1: Heme-oxygenase-1
IL: Interleukin
LN: Lupus nephritis
LPS: Lipopolysaccharide
MDSCs: Myeloid-derived suppressor cells
NF-κB: Nuclear factor kappa-light-chain-enhancer of activated B cells
NLRP3: NACHT, LRR, and PYD domain-containing protein 3
NQO-1: NAD(P)H:-quinone oxidoreductase 1
Nrf2: Nuclear factor E2-related factor 2
Pbmc: Peripheral blood mononuclear cell
RLU: Reactive luminescence units
ROS: Reactive oxygen species
SEM: Standard error of the mean
SLE: Systemic lupus erythematosus
