# Supplementary material for: Baicalein ameliorates pristane-induced lupus nephritis via activating Nrf2/HO-1 in myeloid-derived suppressor cells
Source: Arthritis Res Ther. 2019 Apr 25;21:105. doi: 10.1186/s13075-019-1876-0 (PMC6482536; doi:10.1186/s13075-019-1876-0)
Supplement: Supplementary file 1 — Figure S1. Baicalein attenuates inflammation of lung and joint in pristane-induced lupus mice. BALB/c WT mice (n = 7/group) were given a single injection of 0.5 ml pristane and kept for 5 months. Then mice were randomly divided into three groups: vehicle, 25 mg/kg baicalein, 100 mg/kg baicalein and kept for another 2 months. (A) Lung sections from each groups showed histologic differences. (B) Representative histological sections of tarsal hind paw joints showing normal appearance and severe inflammatory infiltration and bone loss. Data represent the mean scores ± SEM. *P ≤ 0.05, **P ≤ 0.01, ***P ≤ 0.001. n = 7 animals per group. Figure S2. Baicalein attenuates the serum level of pro-inflammatory cytokines in lupus mice. (A) The level of IFN-γ in serum. (B) The level of IFN-α in serum. (C) The level of IL-17A in serum. (D) The level of IL-6 in serum. Data represent the mean scores ± SEM. *P ≤ 0.05, **P ≤ 0.01, ***P ≤ 0.001. n = 7 animals per group. Figure S3. Baicalein reduces the expansion of inflammatory cell in pristane-induced lupus mice. (A) The percentage of total T cells in spleen. (B) The percentage of activated T cells in spleen. (C) The percentage of activated B cells in spleen. (D) The percentage of macrophage cells in spleen. (E) The percentage of mature dendritic cells in spleen. (F) The percentage of macrophage cells in kidney. Data represent the mean scores ± SEM. *P ≤ 0.05, **P ≤ 0.01, ***P ≤ 0.001. n = 7 animals per group. Figure S4. The effect of baicalein on MDSCs apoptosis. (A) BM cells from 6-8w female mice were cultured for 4 days with GM-CSF (40 ng/ml) and IL-6 (40 ng/ml), the proportions of CD11b+Gr-1+ MDSCs were analyzed by flow cytometry. (B) The statistical results of the frequency of MDSCs. (C) MDSCs were treated with BA (0.01 μM, 0.02 μM, 0.04 μM, 0.06 μM) for 24 h and the apoptosis cells were detected with Annexin V by flow cytometry. (D) The cell viability was determined by a CCK8 assay. (E) The cell cycle was determined by flow cytome [file 13075_2019_1876_MOESM1_ESM.docx]

**Supplementary materials and methods**

**
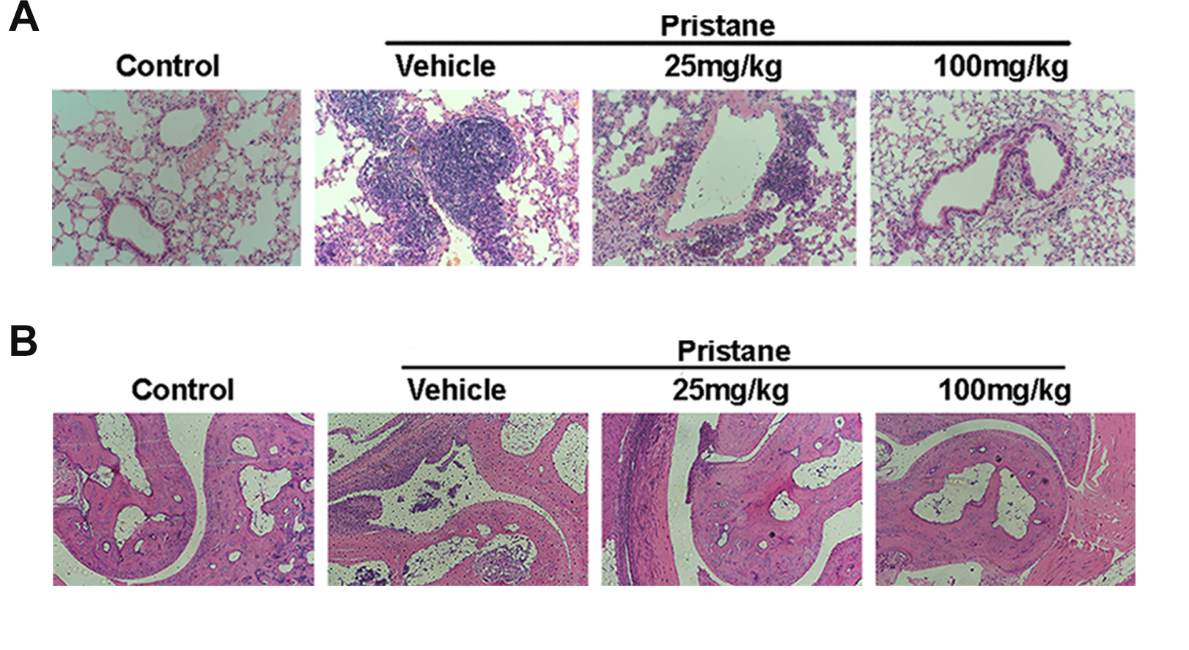
**

**Figure S1. Baicalein attenuates inflammation of lung and joint in pristane-induced lupus mice.** BALB/c WT mice (n=7/group) were given a single injection of 0.5ml pristane and kept for 5 months. Then mice were randomly divided into three groups: vehicle, 25mg/kg baicalein, 100mg/kg baicalein and kept for another 2 months. (A) Lung sections from each groups showed histologic differences. (B) Representative histological sections of tarsal hind paw joints showing normal appearance and severe inflammatory infiltration and bone loss. Data represent the mean scores ± SEM. *P≤0.05, ** P≤0.01, *** P ≤0.001. n = 7 animals per group.


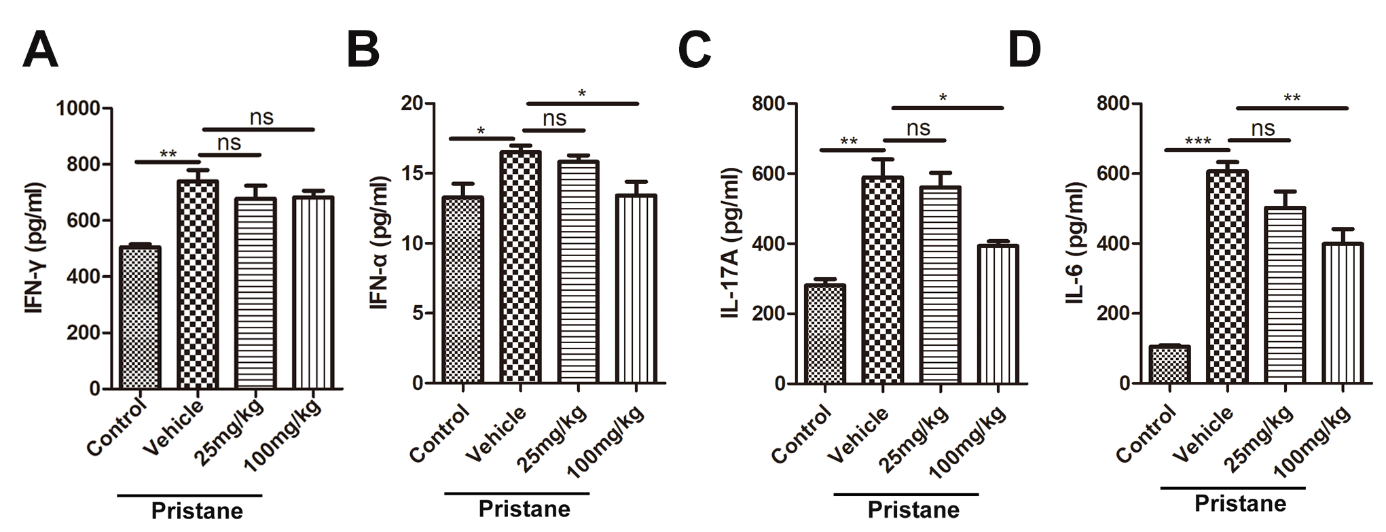


**Figure S2. Baicalein attenuates the serum level of pro-inflammatory cytokines in lupus mice.** (A) The level of IFN-γ in serum. (B) The level of IFN-α in serum. (C) The level of IL-17A in serum. (D) The level of IL-6 in serum. Data represent the mean scores ± SEM. *P≤0.05, ** P≤0.01, *** P ≤0.001. n = 7 animals per group.


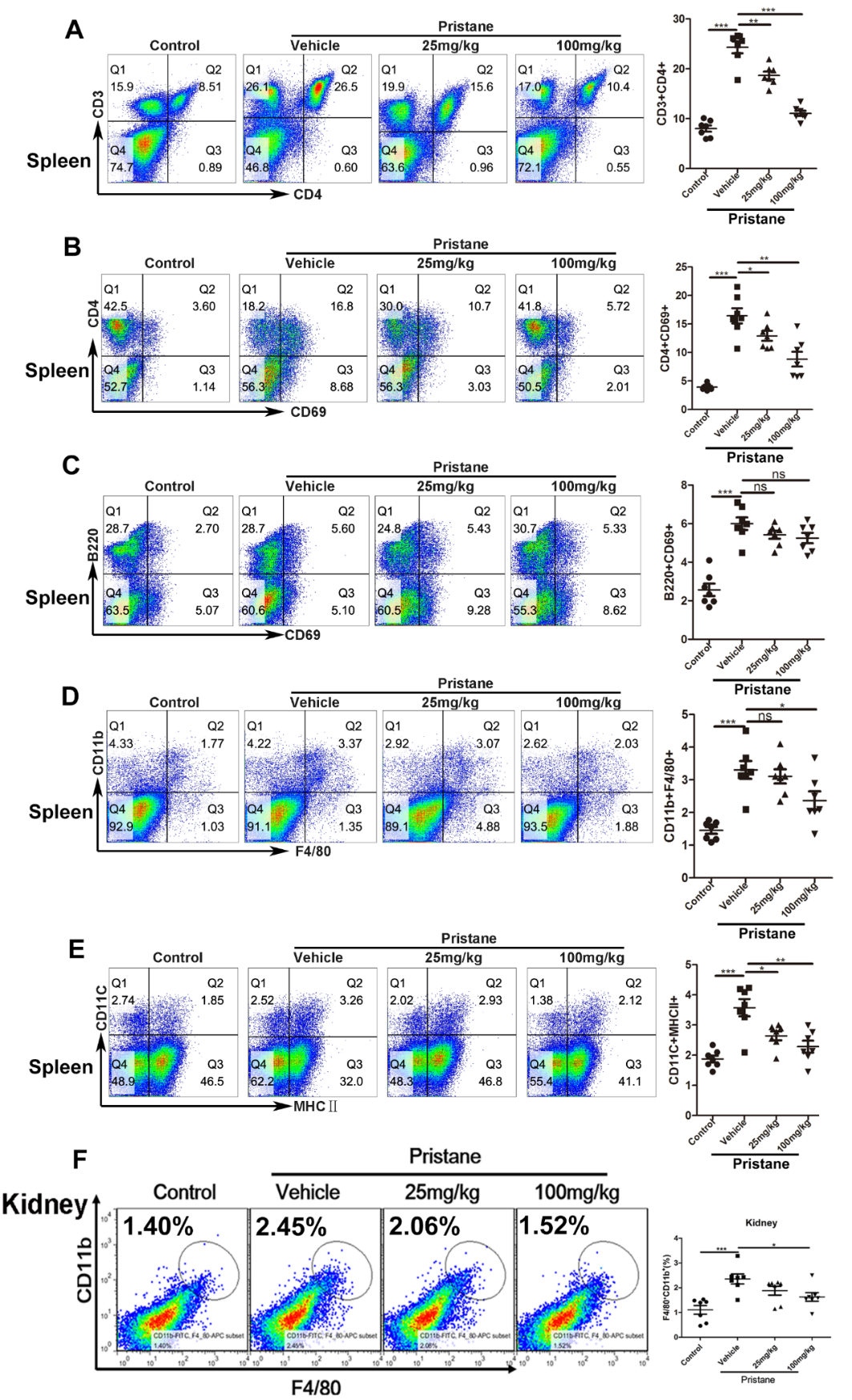


**Figure S3. Baicalein reduces the expansion of inflammatory cell in pristane-induced lupus mice.** (A) The percentage of total T cells in spleen. (B) The percentage of activated T cells in spleen. (C) The percentage of activated B cells in spleen. (D) The percentage of macrophage cells in spleen. (E) The percentage of mature dendritic cells in spleen. (F) The percentage of macrophage cells in kidney. Data represent the mean scores ± SEM. *P≤0.05, ** P≤0.01, *** P ≤0.001. n = 7 animals per group.


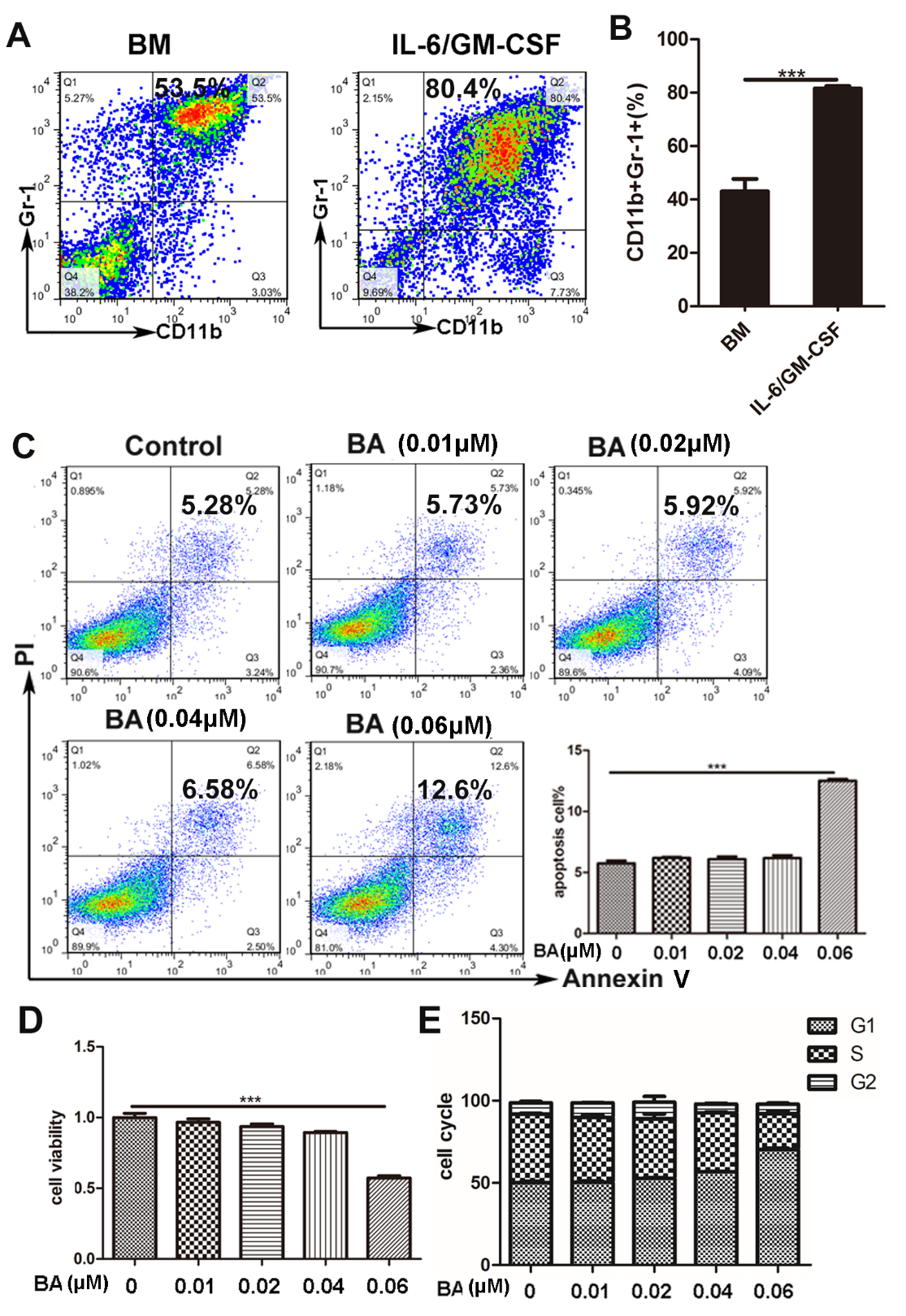


**Figure S4. The effect of baicalein on MDSCs apoptosis.** (A) BM cells from 6-8w female mice were cultured for 4 days with GM-CSF (40ng/ml) and IL-6 (40ng/ml), the proportions of CD11b^+^Gr-1^+^ MDSCs were analyzed by flow cytometry. (B) The statistical results of the frequency of MDSCs. (C) MDSCs were treated with BA (0.01 µM, 0.02 µM, 0.04 µM, 0.06

µM) for 24 h and the apoptosis cells were detected with Annexin V by flow cytometery. (D) The cell viability was determined by a CCK8 assay. (E) The cell cycle was determined by flow cytometery. Data represent the mean scores ± SEM of triplicate experiments. *P≤0.05, ** P≤0.01, *** P ≤0.001.

**Table S1. Primers of mouse gene used for real-time RT-PCR**

| **Gene** | **Forward primer (5’-3’)** | **Reverse primer (5’-3’)** |
| --- | --- | --- |
| iNOS | CCAAGCCCTCACCTACTTCC | CTCTGAGGGCTGACACAAGG |
| ARG-1 | CTCCAAGCCAAAGTCCTTAGAG | GGAGCTGTCATTAGGGACATCA |
| p47^phox^ | AGAACAGAGTCATCCCACAC | GCTACGTTATTCTTGCCATC |
| gp91^phox^ | TCACATCCTCTACCAAAACC | CCTTTATTTTTCCCCATTCT |
| HO-1 | CTGGAAGAGGAGATAGAGC | CTGGTGTGTAAGGGATGG |
| NQO-1 | AACGACATCACAGGGGAG | GCACCCCAAACCAATACA |
| NLRP3 | ATTACCCGCCCGAGAAAGG | TCGCAGCAAAGATCCACACAG |
| IL-1β | GAAATGCCACCTTTTGACAGTG | TGGATGCTCTCATCAGGACAG |
| GAPDH | AGGTCGGTGTGAACGGATTTG | GGGGTCGTTGATGGCAACA |
